# Large-Scale Brain Network Coupling Predicts Total Sleep Deprivation Effects on Cognitive Capacity

**DOI:** 10.1371/journal.pone.0133959

**Published:** 2015-07-28

**Authors:** Yu Lei, Yongcong Shao, Lubin Wang, Tianye Zhai, Feng Zou, Enmao Ye, Xiao Jin, Wuju Li, Jianlin Qi, Zheng Yang

**Affiliations:** 1 Beijing Institute of Basic Medical Sciences, Beijing, PR China; 2 Cognitive and Mental Health Research Center, Beijing, PR China; 3 Department of Clinical Psychology, Air-Force General Hospital, Beijing, PR China; National Scientific and Technical Research Council (CONICET), ARGENTINA

## Abstract

Interactions between large-scale brain networks have received most attention in the study of cognitive dysfunction of human brain. In this paper, we aimed to test the hypothesis that the coupling strength of large-scale brain networks will reflect the pressure for sleep and will predict cognitive performance, referred to as sleep pressure index (SPI). Fourteen healthy subjects underwent this within-subject functional magnetic resonance imaging (fMRI) study during rested wakefulness (RW) and after 36 h of total sleep deprivation (TSD). Self-reported scores of sleepiness were higher for TSD than for RW. A subsequent working memory (WM) task showed that WM performance was lower after 36 h of TSD. Moreover, SPI was developed based on the coupling strength of salience network (SN) and default mode network (DMN). Significant increase of SPI was observed after 36 h of TSD, suggesting stronger pressure for sleep. In addition, SPI was significantly correlated with both the visual analogue scale score of sleepiness and the WM performance. These results showed that alterations in SN-DMN coupling might be critical in cognitive alterations that underlie the lapse after TSD. Further studies may validate the SPI as a potential clinical biomarker to assess the impact of sleep deprivation.

## Introduction

Sleep deprivation (SD) or sleep loss is ordinary in modern society. The increasing time of sleep loss could lead to sleepiness, involuntary microsleep, problems in sustained attention, and cognitive slowing [1–3]. However, the mechanism underlying the effects of sleep deprivation on cognitive capacities still remains to be elucidated. According to an emerging energy allocation (EA) model, biological processes that remain unfulfilled to sleep loss largely lead to the functional deficits[4]. The longer a person remains awake, the greater the tension between the mounting homeostatic pressure for sleep and motivated attempts to fight off sleep, leading to increased fluctuations in alertness. This is known as the ‘wake state instability’ hypothesis[5,6]. Though both of the theories are prominent, none of them involves the neural mechanism of sleep deprivation.

The science of large-scale brain networks offers a powerful paradigm for investigating cognitive dysfunction after sleep deprivation, suggesting that cognitive deficits induced by SD are characterized by multiple brain areas involving several distinct brain systems. A neuroimaging study demonstrated that sleep deprivation could lead to reduced metabolic activity within a network of brain regions important for information processing and executive control, including the prefrontal cortex, anterior cingulate, thalamus, and cerebellum[7]. More recently, Drummond and colleagues showed that a cortical network important for sustained attention was altered during sleep deprivation[8]. According to these studies, Namni and colleagues proposed that two competing neuro-biologic systems might have an influence on the behavior of a sleep-deprived individual. The top-down drive to maintain alertness is generated from more rostral areas of the brain, while the involuntary homeostatic drive to fall asleep comes from more central and caudal areas[9]. Furthermore, Killgore suggested that abnormal activation of default mode network (DMN) induced by sleep deprivation might result in a failure to effectively allocate resources to task-relevant brain regions[6]. Inspired by recent developments in large-scale brain network analysis, researches in sleep deprivation found that SD was associated with reduced functional connectivity of main DMN and anti-correlation network (ACN) nodes during rest[10,11]. However, these studies were limited to bivariate correlations between node pairs. Consequently, SD-induced changes of network interaction could not be fully detected.

Of the many intrinsic connectivity networks (ICN) identified in the human brain, three have received the most attention: an executive control network (ECN) implicated in a wide range of cognitively demanding tasks; a default mode network typically deactivated during most stimulus-driven cognitive tasks; and a salience network (SN) involved in detecting, integrating, filtering external vs internal stimuli and allocating attention[12–19]. Emerging evidence suggests that SN, an integral hub in regulating dynamic interactions between other large-scale brain networks, plays an important role in initiating network switching leading to the engagement of the ECN and the disengagement of the DMN[14,15,20]. In conclusion, Menon proposed a triple network model which focused on the SN, DMN, and ECN, indicating that dysfunction in one network can impact the other two networks[20]. Triple network model provides a new insight to study dysfunction in psychopathology, such as autism, major depression, obesity, and dementia, revealing that characterization of the SN and its interaction with the DMN and ECN is an important aspect of deficits in cognitive function [21–25]. In a recent study in nicotine dependence, Lerman and colleagues found that alterations in SN-DMN coupling and the inability to disengage from the DMN might be critical in cognitive/affective alterations that underlie nicotine dependence[26].

The goal of this work was to explore the effect of sleep deprivation on the interaction within large-scale brain networks underlying the ‘wake state instability’ hypothesis and EA model. In this work, we applied data-driven independent component analysis (ICA) to the resting state functional magnetic resonance imaging (fMRI) data to extract ICNs, as ICA has turned out to be a useful tool for large-scale network analysis, reflecting strong coupling of spontaneous fluctuations in ongoing activity and remaining robust under different mental states[27–29]. More importantly, ICNs identified by ICA are less sensitive to physiological noise and other artifacts, offering robust way to characterize large-scale brain organization[12,30]. Given that the SN plays an important role in toggling resources between the ECN and DMN, a quantitative network association index integrating the SN and DMN is proposed in this work to assess sleep pressure during sleep deprivation, referred to as sleep pressure index (SPI). In the present study, we examined the SPI in individuals undergoing functional magnetic resonance imaging in rested wakefulness (RW) and after 36 h of TSD. Moreover, we tested the hypothesis that the SPI would (1) be stronger in the individuals after TSD, (2) have significant correlation with individual’s sleepiness state, and (3) predict cognitive performance in working memory (WM) task.

## Materials and Methods

### Participants

Fourteen healthy and right-handed adult males with normal or corrected-to-normal vision were recruited from Beijing Normal University as paid volunteers by advertisements. None of the subjects had previously participated in psycho-physiological experiments. Exclusion criteria were diseases of the central and peripheral nervous systems, head trauma, cardiovascular diseases and/or hypertension, cataracts and/or glaucoma, pulmonary problems, or alcohol or drug abuse. Written informed consent according to the Declaration of Helsinki was obtained from each of the subjects after a complete description of the study. Participants were instructed to habitually keep a regular sleep schedule and refrain from alcohol, caffeine, chocolate intake and napping for 1 week before the study and during it in order to establish typical sleep patterns, defined as 8 h of sleep per night. This study was approved by the Research Ethics Committee of Beijing Institute of Basic Medical Sciences and the Fourth Military Medical University (Xi’an, China).

### Experiment Paradigm

The experiment was conducted in the Basic Aerospace Institute (Beijing, China). Nursing staff monitored subjects 24 hour a day and each subject was assigned a partner to keep them awake through the night while under continuous behavior monitoring. During the TSD session, participants were allowed to do some non-strenuous activities such as reading and talking with their partners and not allowed to leave the lab until they were escorted to the fMRI facility.

The participants were scanned both during RW and after 36 h of TSD. Half participants started with the RW session; the remaining participants started with the TSD session to reduce the potential influence of scan order. Considering the possibility of residual effects of TSD, the two scanning sessions were conducted 3 weeks apart and performed at the same time (8:00 PM). After each scan session, the visual analogue scales (VAS) were obtained to evaluate subject’s alertness and mood state. In this work, the VAS of alertness, anxiety, energy, self-confidence, irritability, nervousness, sleepiness and talkativeness were measured by eight 10 cm lines, yielding scores that range from 0 to 10. Participants were required to mark the line with their current subjective rating.

### Working Memory Task Paradigm

A visual N-back WM task lasting 8 minutes and 24 seconds was employed to characterize the impact of 36 h TSD on behavior performance. The N-back task involved presentation of letter figures for 400msec, followed by fixation stimulus for 1600msec under three conditions: 0-back, 1-back, and 2-back. In the 0-back condition, participants responded with a button press to a specified target. For the 1-back condition, participants responded if the current letter was identical to the previous one. In the 2-back condition, participants responded if the current letter was identical to that two trials back. Each condition consisted of 18 trials (36-sec block) and the 0-back condition was repeated 7 times, while each of the other two conditions was repeated 3 times. In the preliminary experiment, we also employed 3-back WM task. However, considering the much too low accuracy of 3-back condition due to the lapse of behavior performance after 36 h TSD, we only employed the above three conditions in this study.

### Functional Magnetic Resonance Imaging Data

Imaging acquisition was performed at the General Hospital of the PLA of China on a GE 3.0T Signa scanner (General Electric Medical System, Milwaukee, WI) with a birdcage RF imaging coil. Structural MRI and fMRI scanning including resting-state procedures and working memory tasks were performed on each participant. Participants were asked to lie in the scanner with their head comfortably restrained to reduce head movement. During the resting-state scans, subjects were instructed to relax and keep their eyes closed, remain as motionless as possible, and not to think of anything in particular. In order to record participant’s cardiac activity during scanning, a pulse oximeter was attached to the participant’s finger. In addition, participants wore a pressure belt around the abdomen to record their respiratory activity. The cardiac and respiratory signals were collected and synchronized to the fMRI data in order to remove these physiologic variations during the regression analysis.

In the resting-state scan session, an echo-planar imaging (EPI) sequence was used to collect 189 functional volumes with following parameters: echo time (TE), 30 ms; repetition time (TR), 2000 ms; field of view, 256 × 256 mm; slice thickness, 5 mm; slice gap, 1 mm; flip angle (FA), 90°; matrix size, 64 × 64; 20 oblique slices. A high-resolution T1-weighted anatomical image was acquired using an SPGR sequence. In order to keep the subjects awake during the scan, they were reminded to stay awake through the microphone before each run. After each run, all the subjects confirmed that they were awake in the previous run.

### FMRI Data Analysis

#### Preprocessing

FMRI data was preprocessed using Analysis of Functional Neuro Images (AFNI) software (AFNI, http://afni.nimh.nih.gov/afni/). The high-resolution anatomical images were first manually transformed into the standard Talairach space. For fMRI image preprocessing, the initial 10 volumes of resting-state datasets were discarded to allow for the equilibration of the magnetic resonance imaging signal and subjects’ adaptation to the scanning noise. Cardiac and respiratory noises were regressed out by 3dretroicor in AFNI. As one subject’s physiological data was incomplete, the final dataset included 13 subjects for further analysis. This was followed by despiking (compression of extreme time series outliers using a hyperbolic tangent function, 3dDespike in AFNI), slice timing (3dTshift in AFNI), volume registration, motion correction (3dvolreg in AFNI), quadratic detrending (3dDetrend in AFNI), spatial smoothing (Gaussian kernel of full width at half maximum of 6mm, 3dmerge in AFNI) and manual normalization to Talairach space with a resampled resolution of 3×3×3 mm^3^ (adwarp in AFNI).

#### Independent Component Analysis of FMRI Data

We applied group probabilistic independent component analysis (GICA) to the resting data using FSL MELODIC (FMRIB Analysis Group, Oxford University)[31,32]. The preprocessed data from both groups in Talairach space were submitted to MELODIC using the command-line tool with the component number set at 30 and the decomposition approach set as temporal concatenation. The GICA spatial maps were converted to z score maps and then thresholded via a mixture model fit (p > 0.5) to identify voxels contributing to each independent component. The SN, DMN, and ECN were identified by visual inspection of the thresholded GICA maps.

A previously described and validated dual-regression procedure was applied to generate a time course for each component[33]. Dual-regression is a statistical approach that can be applied after GICA and involves a first regression that uses group-level spatial components to find time courses associated with each component in each individual, followed by a second regression that uses the individual time courses to find subject-specific spatial maps for each component. As a measure of cross-network coupling, we calculated absolute values of Pearson correlation coefficients (CC) between component time courses derived from the SN, ECN, and the DMN (CC_SN, DMN_ and CC_SN, ECN_) to represent the coupling strength between different networks. Then, Fisher’s z transformation was applied to the CC values, yielding variants of approximately normal distribution [z = 0.5 ln (1 + r)/(1 - r)].

#### Correlation between network couplings and behavioral data

Firstly, we used Pearson Correlation to investigate the relationship between SN-DMN coupling strength and VAS score of sleepiness. Then, working memory task for each subject was evaluated by both the average percent correct (PC) and reaction time (RT) on the 2-back verbal working memory task. RT was determined using only trials with correct responses, excluding lapse trials (no response or incorrect response). Given that the SN is an integral hub in initiating network switching between other large-scale brain networks, we correlated both SN-DMN and SN-ECN (left ECN and right ECN) coupling strength across subjects with working memory task performance to investigate the relationship between behavior performance and large-scale network couplings. In the above correlation analysis between behavioral data and network couplings, all the data were demeaned within each group to remove the mean group effect

## Results

### Descriptive Data

The fourteen participants had a mean age of 25.9 years (standard error, 2.3 years; range, 21–27 years). None of the subjects showed evidence of clinical symptom levels as assessed by the Symptom Checklist-90 (SCL-90) with T-scores <60 on the General Symptom Index, and all the participants had normal intelligence scores (Raven test, intelligent quotient [IQ] >100).

During the fMRI scanning, all subjects’ respiration and heart rates were monitored. The mean values of individual respiration and heart rates before and after SD were compared using paired t-test. No differences were found in heart or respiratory rate between the RW and TSD conditions (Heart rate: RW 68.42 ± 7.26, TSD 72.00 ± 6.61, t[1, 13] = -1.500, p = 0.161; Respiratory rate: RW 19.01 ± 2.42, TSD 18.42 ± 2.62, t[1, 13] = -1.084, p = 0.300). No one was excluded for head movement, exceeding more than 1mm translational movement or more than 1° rotational movement. No significant differences were found in the head movement between the RW and TSD conditions (Paired t test, p = 0.463).

### Resting Networks

**Fig 1:**
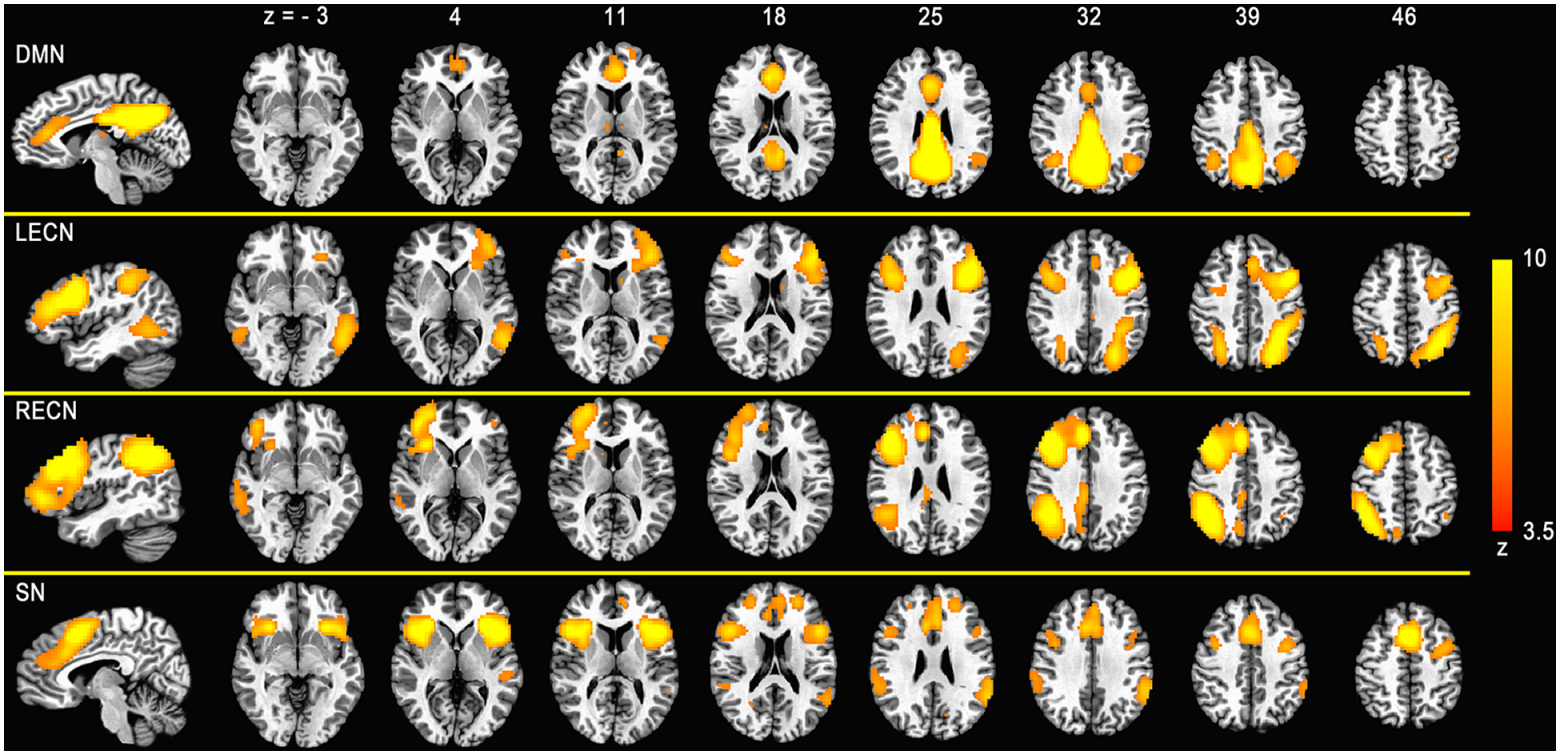
Group-level components revealed by the temporal concatenation group independent component analysis (ICA). Four networks generated from group ICA of the resting state data were identified as the default mode network (DMN), left and right executive control network (ECN) and salience network (SN). Spatial maps were converted to z score images and then thresholded at z = 3.5 via mixture model fit. Network maps are displayed in red-yellow overlaid onto the Talairach space based on radiological convention (left = right).

In the 30 GICA components, four were identified as SN, ECN, and DMN and were thresholded for display at z = 3.5 (Fig 1). As an observation commonly reported, the ECN was strongly lateralized and referred to as the left ECN (LECN) and right ECN (RECN). Spatial cross-correlation between these networks and previously defined maps [34] revealed a high degree of similarity (r(DMN) = 0.48, r(LECN) = 0.35, r(RECN) = 0.43, r(SN) = 0.45). In addition, other canonical functional networks (dorsal attention network [DA], primary visual cortex network [PVC], second visual cortex network [SVC], auditory cortex network [AC], sensorimotor network [SM]) were identified from the 30 GICA components (S1 Fig).

### Shifted Intrinsic Connectivity

**Fig 2:**
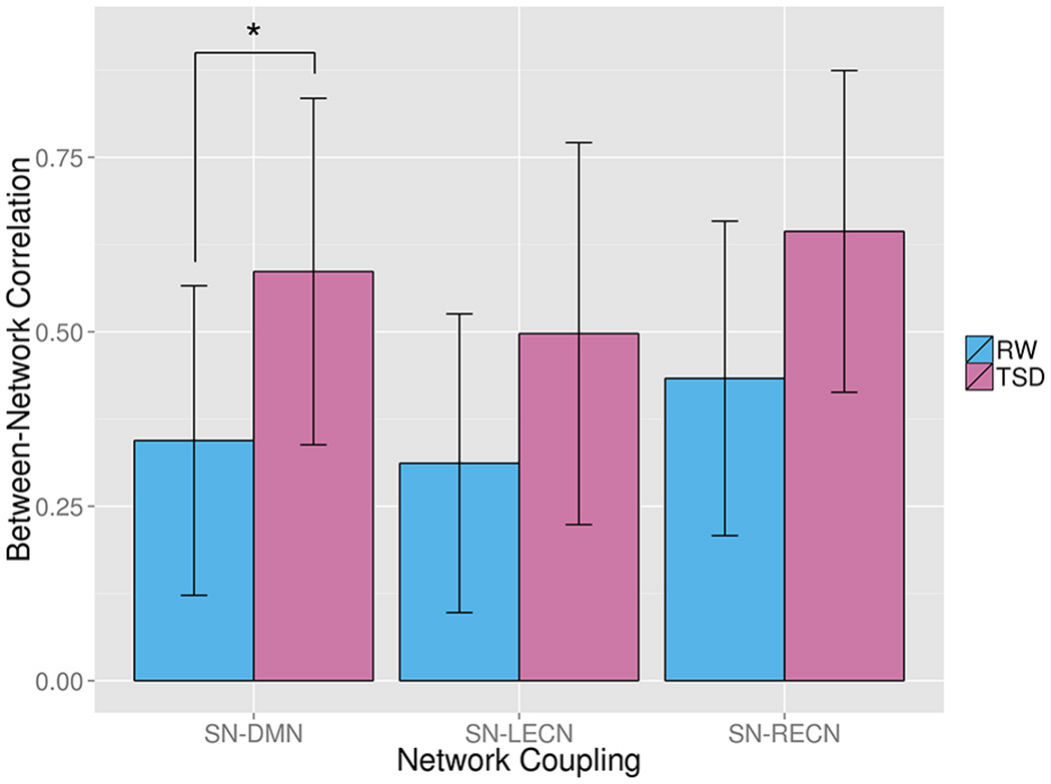
Significant increase in correlation between the salience network (SN) and the default mode network (DMN) (paired t-test, p = 0.0012) in the TSD vs RW states. LECN indicates left executive control network; RECN, right executive control network. *p < 0.01, statistical significance.

To explore the shifted intrinsic connectivity across SN, ECN, and DMN, we tested the significance of group differences of pairwise correlations between network time courses using paired two-sample t-test (Fig 2). In TSD group, CC_SN, DMN_ was significantly increased, compared with that in RW group (paired t-test, p = 0.0012). The changes in CC_SN, LECN_ and CC_SN, RECN_ showed an increased trend in TSD group as well, while they were not significant (paired t-test, p < 0.01). We also checked the changes of functional coupling between SN and other canonical networks including DA, PVC, SVC, AC and SM after 36 h of TSD. The results showed that the functional couplings between SN and AC, DA, PVC and SM were significantly increased after 36 h of TSD (paired t-test, p < 0.01) (S2 Fig). However, correlation analysis showed that there were no significant correlations between these functional couplings and behavioral data.

### Correlation Between Network Couplings and Sleepiness

**Fig 3:**
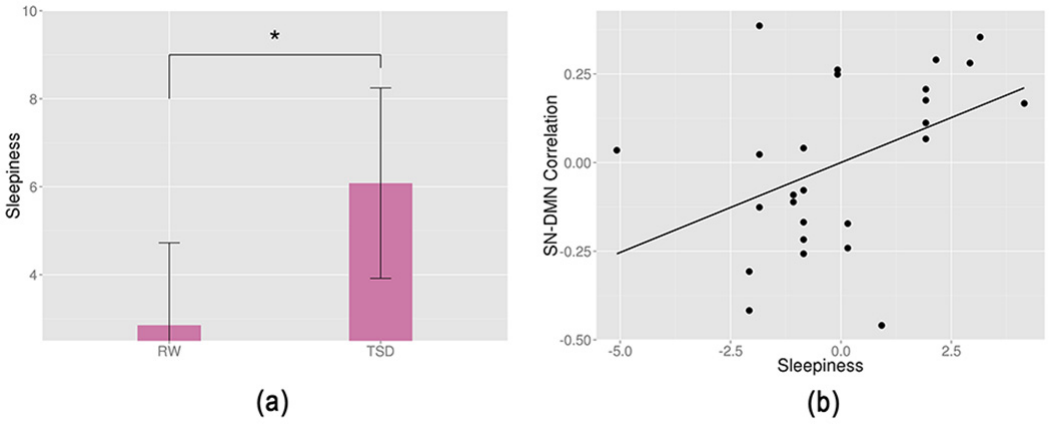
(a) Significant increase in sleepiness score after 36 h of TSD (paired t-test, p = 0.00011). (b) Regression plots of correlation between the salience network (SN) and the default mode network (DMN) against sleepiness score. *p < 0.01, statistical significance.

In this study, VAS was used to measure sleepiness and mood state of each subject (Table 1). The results showed that participants were sleepier after 36 h of TSD (paired t-test, p < 0.01, Fig 3a). In addition, we also explored the relationship between sleepiness scores and large-scale brain network couplings. The results showed that there was a positive trend for an association between the SN-DMN coupling and sleepiness score (r = 0.436, p = 0.026), whereas neither SN-LECN nor SN-RECN was significantly correlated with alertness score (Fig 3b).

**Table 1:** Behavioral data in RW state and after 36 h of TSD.

Scale/task	RW	TSD
Visual analogue scales		
Factor 7 (sleepiness)	7.62 (1.0)	5.62 (1.64)
2-back working memory task		
Percent correct	1.506 (0.275)	1.38 (0.303)
Reaction time (s)	0.689 (0.139)	0.765 (0.189)

### Correlation Between Network Couplings and Working Memory Performance

**Fig 4:**
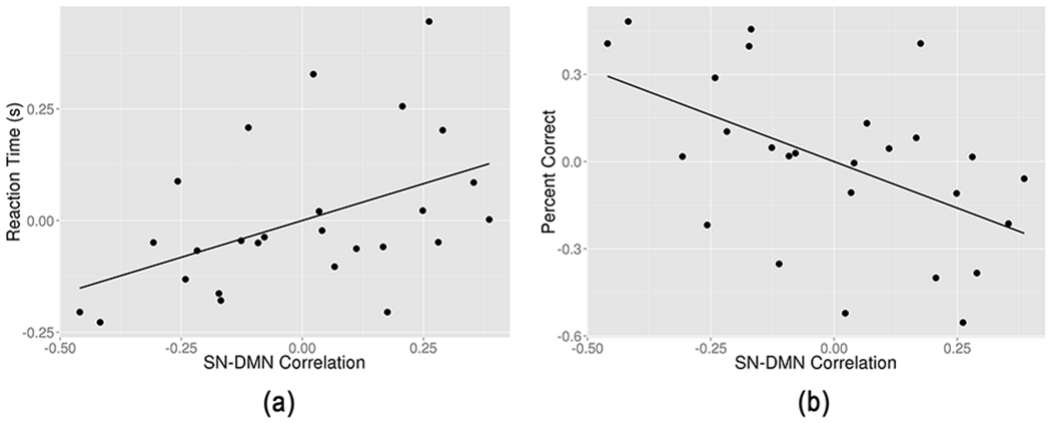
Regression plots of correlation between the salience network (SN) and the default mode network (DMN) against (a) the reaction time and (b) the percent correct of 2-back working memory (WM) task.

Relative to RW, responses in TSD were slower and less accurate (Table 1). Partially significant correlation was observed between SN-DMN coupling and RT (Fig 4a). The increase of the SN-DMN coupling predicted longer RT in WM task (r = 0.467, p = 0.016). We also found a negative trend for an association between SN-DMN coupling and WM task percent correct (PC, r = -0.5197, p = 0.0065) (Fig 4b); as the SN-DMN coupling increased, the participant responded more incorrectly. In addition, both SN-LECN and SN-RECN were correlated with WM cognitive performance (either RT or PC), while no significance was found (p < 0.01). Both the longer RT and decreased PC revealed that SN-DMN was a reliable index to predict the WM cognitive performance.

## Discussion

When a person remains awake longer, the biological pressure for sleep will become stronger. According to the EA model, during prolonged sleep deprivation, the biological investment (BI), defined as the energy needed for all other biological activities not requiring either vigilance or motor activity, was upregulated via reactive homeostasis during the waking state to enhance survival[4]. When the time of attempting to sustain wakefulness exceeds 36 h, the balance between the mounting homeostatic pressure for sleep and motivated attempts to fight off sleep will be broken. Depending on the drive of sleep increases or decreases over certain thresholds, sleep is triggered or otherwise wakefulness would occur[9]. Studies indicated that prefrontal executive regions played a crucial role in modulating the motivational control over the waking state via top-down cortical control system, while brain stem and hypothalamic nuclei generated the involuntary drive for sleep from bottom-up system [35]. Therefore, alterations in coupling between these networks increase the instability of waking state. In this study, we extend these observations by demonstrating a composite network association index integrating the SN and the DMN. The predictive validity of this novel index was validated by self-reported behavioral data and working memory task performances. The SPI was significantly stronger in subjects after 36 h of TSD, suggesting an inappropriate relationship between SN and DMN or a failure to allocate resources to task-relevant brain regions when needed. Consistently, SPI values significantly correlated with individual’s sleepiness state and stronger SPI values also predicted poorer WM task performance. In conclusion, this index reflects the biological pressure for sleep during sleep deprivation, which could be used to assess the sleep pressure during TSD.

These findings further our understanding of aberrant neural mechanisms underlying cognitive deficits observed in individuals with TSD. Homeostatic pressure for sleep increases against with individual’s motivation to stay awake, reflecting on the interaction of neurobiological systems, one attempting to keep individuals awake and the other driving individuals to fall asleep[9]. At last, lapses develop from momentary periods of inattention to another state that can be described as a functional sleep attack. In neural system level, this lapse can be presumed as a result of the imbalanced functional link between SN-DMN and SN-ECN, in other word, a bias toward enhanced SN-DMN connectivity. Consistent with this assumption, TSD-induced imbalance was observed in this study, characterized by the significantly increased SN-DMN coupling after TSD and non-significant change in SN-ECN correlation. We proposed that in the sleep loss state, SN increased the allocation of attentional resources to fight against the increasing biological pressure for sleep, leading to an enhanced SN-ECN connectivity. In other words, after TSD, much increased SN-DMN coupling revealed the upregulated assignment of saliency to internal mental events for survival while SN-ECN also increased slowly to meet demands of longer waking state. The biased resource allocation toward DMN proved that homeostatic process for survival was priority during sleep deprivation.

In this work, we found significantly positive correlation between SPI and self-reported sleepiness score. As SN plays an important role in switching between external stimuli and internal mental events, increased SPI means that more resources are assigned to self-referential mental events, leading to deficits in alertness, vigilance and attention. In addition, the strong correlation between changes in the SPI and changes in RT or PC suggests that alteration of SPI may be attributable to the detection of salient events and modulation of activity via the SN. In WM task, faster responses were associated with activation in ECN; in contrast, slow responses were associated with even greater activation of medial prefrontal regions implicated in the DMN[8]. Since a role for the SN in modulating relative activity in the ECN vs DMN, the positive correlation between SPI and RT is consistent with the aberrantly increased activation of DMN, which may lead to a failure to effectively allocate resources to task-relevant brain regions in ECN. In addition to RT, a negative correlation between SPI and PC was also observed. This observation is consistent with a recent meta-analysis revealed that sleep deprivation affect not only accuracy but also response time during WM tasks[36]. The robust correlation between SPI and WM task performance indicated the potentiality of SPI to be a biomarker for assessing the impact of sleep deprivation.

In recent network studies, researchers demonstrated that sleep deprivation reduced both DMN connectivity and DMN-ACN correlation during rest[10,11]. They found that increased sleep pressure was reflected in reduced anti-correlation of main DMN and ACN nodes. However, recent brain network studies have pointed out that there are two distinct networks in the ACN, termed as ECN and SN[14]. Our findings of increased coupling in both SN-DMN and SN-ECN after TSD provide compelling parallels to these studies. As DMN is usually deactivated during cognitive tasks, the decoupling between DMN and ACN indicated insufficient allocation of cognitive resources to executive control system, leading to the ‘wake state instability’. In the view of triple network model, the results in this study also revealed the decoupling. When DMN and ECN were negatively coupled well, if the functional coupling between SN and DMN increased, the functional coupling between SN and ECN would decrease. However, the results in this study showed that after 36 h of TSD, the SN-ECN coupling was increased in order to stay awake, nevertheless, the homeostatic pressure for sleep also heavily increased the SN-DMN coupling. This decoupling in allocating cognitive resources leaded to the ‘wake state instability’. Moreover, our study makes much improvement to previous researches. Using ICA and dual-regression analysis, we extracted the time courses of each network while the above two studies just calculated correlation between main node pairs. Another great improvement is the significant correlation between SN-DMN coupling and behavioral measures, in that no correlations between behavioral data and large-scale network couplings were declared in previous sleep deprivation studies.

**Fig 5:**
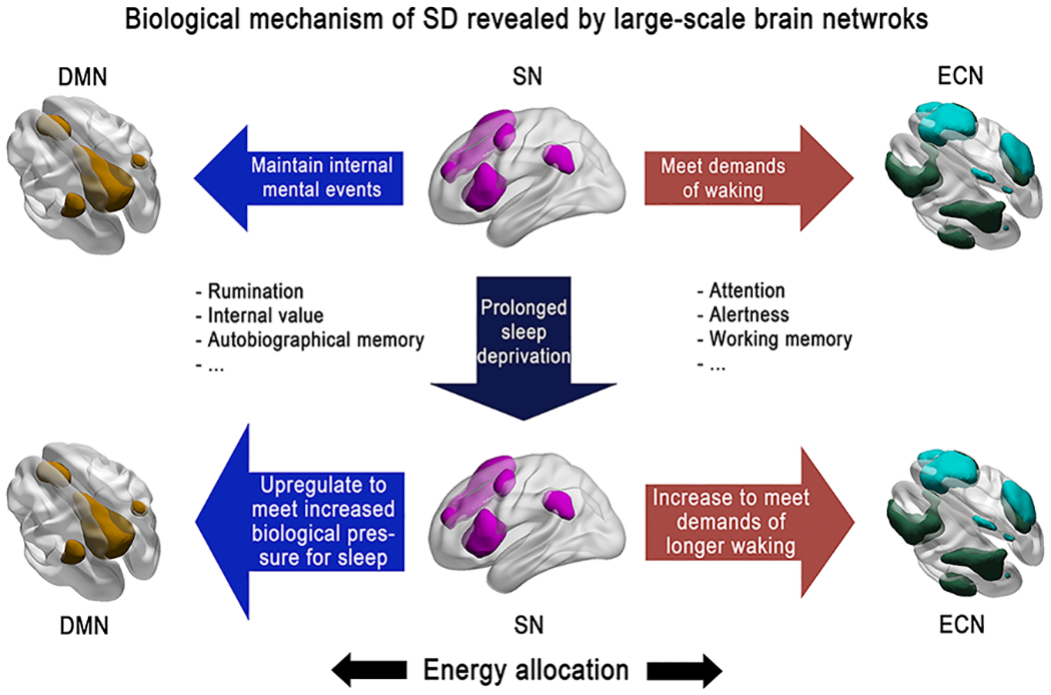
A schematic diagram of sleep deprivation, large-scale brain networks, and energy allocation. In RW state, cognitive resources are appropriately assigned to DMN and ECN in order to meet both internal and external events. With the sleep deprived time increases, though the motivation to remain awake increases, the drive for sleep becomes stronger, leading to lapses at last.

In summary, the present findings point to a novel biological mechanism underlying the lapse after TSD (Fig 5). This study, which is based on the EA model and ‘wake state instability’ hypothesis, furthers our understanding of TSD’s effect from dysregulated DMN to the disability of SN to toggle between ECN and DMN, indicating that SN may play a critical role in cognitive alterations that underlie TSD. On validation, the SPI could serve as a clinical biomarker to assess the impact of sleep deprivation. With additional validation in other cohorts, the SPI could also be a potential biomarker for assessing other cognitive deficits.

## Limitations

Despite the strengths of this study, some limitations should be borne in mind when interpreting these findings. First, if the SPI is to be applied as a diagnostic biomarker, test-retest studies are needed to demonstrate its reproducibility and clinical significance. Second, limitations concerning the use of GICA to identify ICNs should be considered. The data-driven GICA analysis using a 30-component solution was empirical. We also tried GICA using component number of 20 and 25. No significance was found in spatial cross-correlation between the visually identified DMN, SN, and ECN components and those previously published. All of our analyses in this paper were based on network maps with the component number set at 30. Third, considering only male volunteers were assessed in this study due to the experimental conditions (paired subjects) and the long time course of the experiment, we could not generalize our findings to females. Therefore, the clinical utility of the findings will be restricted. In our future study plan, we will investigate gender differences in functional connectivity changes following TSD. Forth, verbal reporting of subjects' in-scanner state is not an adequate way to monitor sleep-deprived participants during the resting state scan [37]. In future experiments, we will consider the use of simultaneous EEG recordings to monitor subjects’ in the scanner. Finally, beyond this triple model, which further brain changes outside the triple network such as subcortical or neurochemical changes are critical for distinct symptoms with sleep deprivation? To disentangle such questions, further studies are needed.

## Conclusions

Alterations in SN-DMN coupling may be critical in cognitive alterations that underlie the lapse after TSD. Further studies may validate the SPI as a potential clinical biomarker to assess the impact of sleep deprivation.

## Supporting Information

S1 Fig Group-level components revealed by the temporal concatenation group independent component analysis (ICA). Five canonical networks generated from group ICA of the resting state data were identified as the dorsal attention network (DA), primary visual cortex network (PVC), second visual cortex network (SVC), auditory cortex network (AC), sensorimotor network (SM). Spatial maps were converted to z score images and then thresholded at z = 3.5 via mixture model fit. Network maps are displayed in red-yellow overlaid onto the Talairach space based on radiological convention (left hemisphere to the viewer’s right).
(TIF)

S2 Fig Significant increase in correlation between the SN and the AC, DA, PVC and SM in the TSD vs RW states (paired t-test, p = 0.01). *p < 0.01, statistical significance.
(TIF)

S1 File The supporting data include 30 GICA components and the corresponding timecourses generated by dual-regression. (ZIP)
