# Supplementary figures and images for: Large-Scale Brain Network Coupling Predicts Total Sleep Deprivation Effects on Cognitive Capacity
Source: PLoS One. 2015 Jul 28;10(7):e0133959. doi: 10.1371/journal.pone.0133959 (PMC4517902; doi:10.1371/journal.pone.0133959)

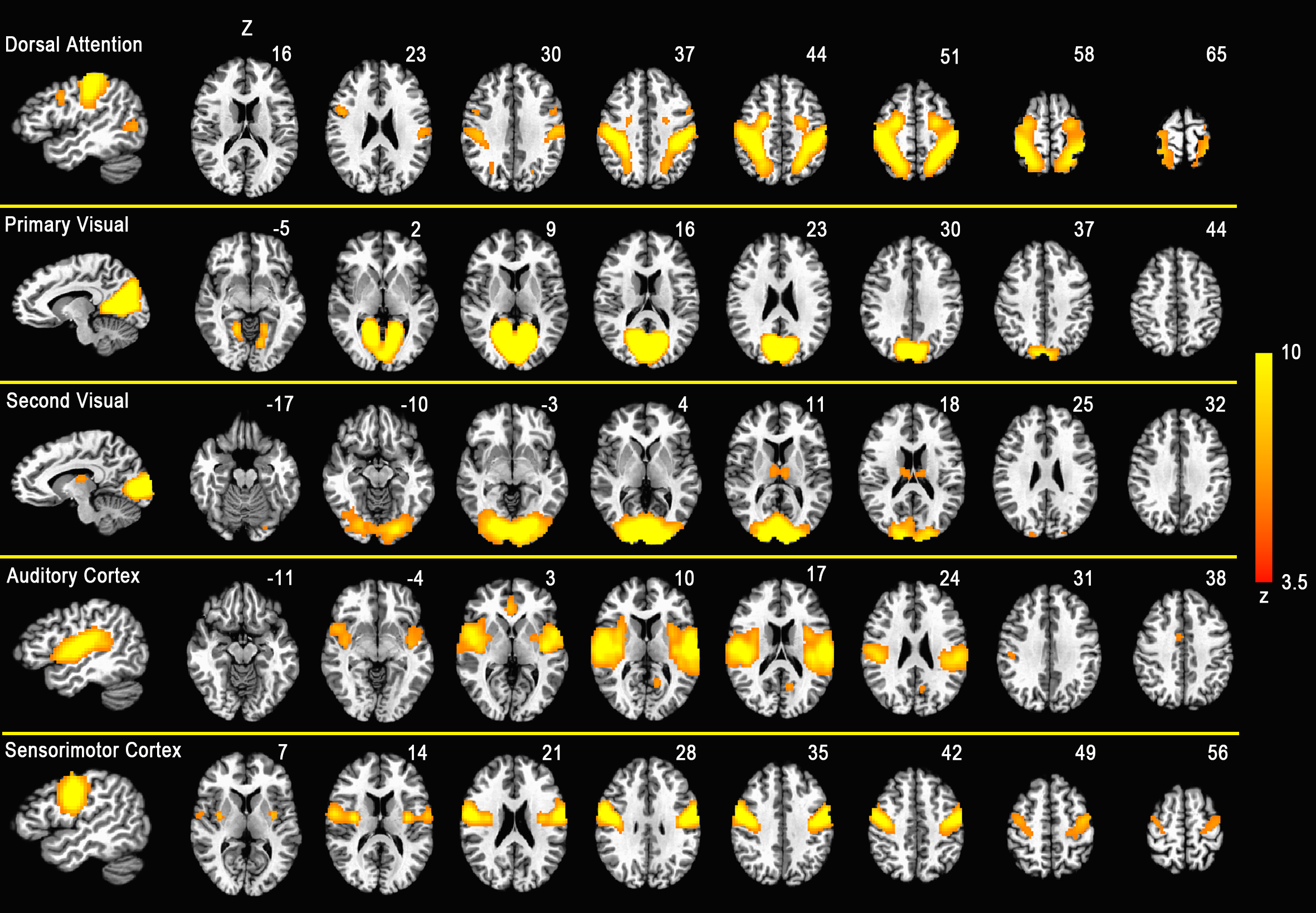

Supplement: S1 Fig — Five canonical networks generated from group ICA of the resting state data were identified as the dorsal attention network (DA), primary visual cortex network (PVC), second visual cortex network (SVC), auditory cortex network (AC), sensorimotor network (SM). Spatial maps were converted to z score images and then thresholded at z = 3.5 via mixture model fit. Network maps are displayed in red-yellow overlaid onto the Talairach space based on radiological convention (left hemisphere to the viewer’s right). (TIF) [file pone.0133959.s001.tif]

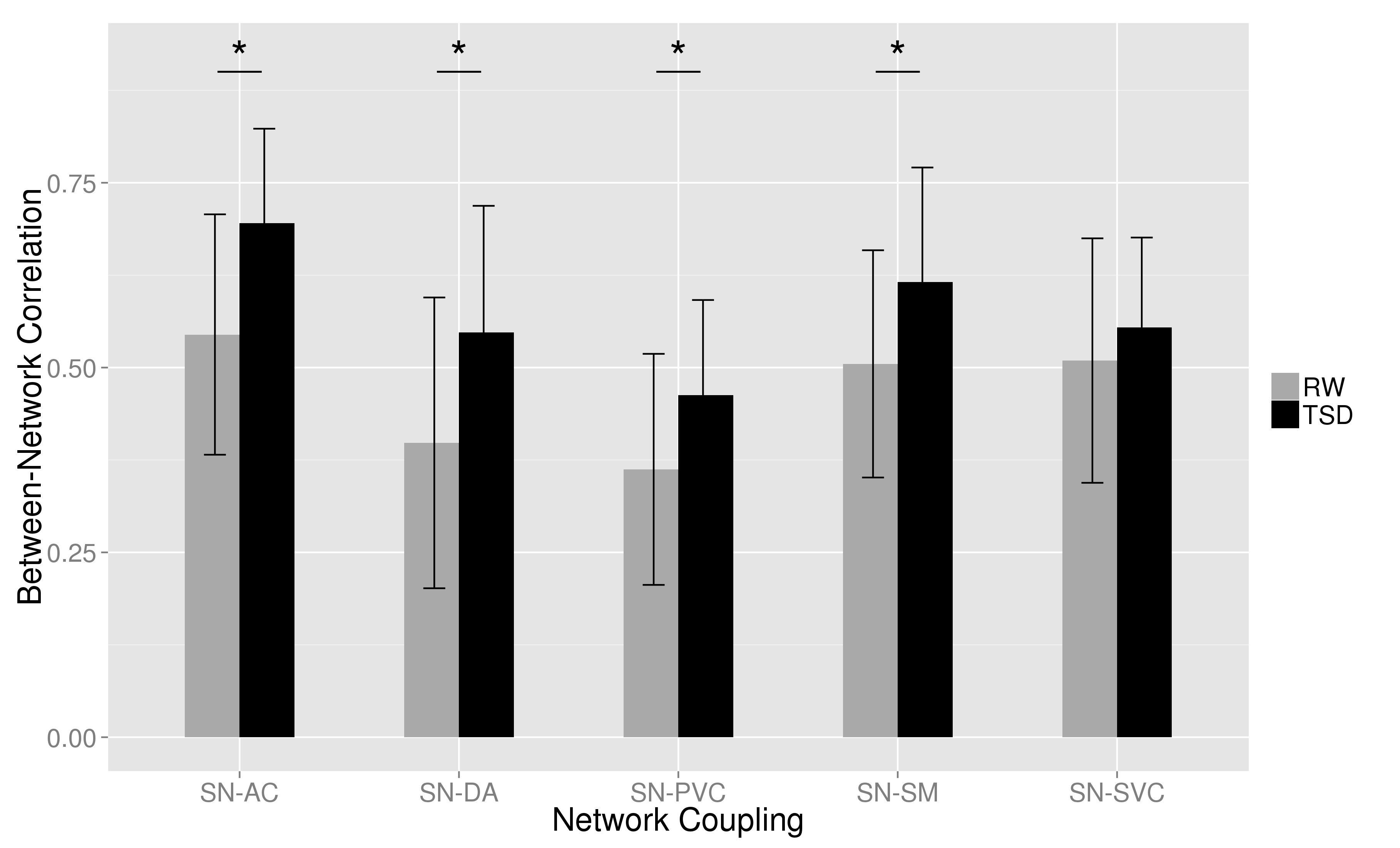

Supplement: S2 Fig — *p < 0.01, statistical significance. (TIF) [file pone.0133959.s002.tif]
